# Cytotoxic and apoptotic effects of Hypericum androsaemum on prostate adenocarcinoma (PC-3) and hepatocellular carcinoma (Hep G2) cell lines with identification of secondary metabolites by LC-HRMS

**DOI:** 10.3906/kim-2104-17

**Published:** 2021-10-19

**Authors:** Nurdan YAZICI BEKTAŞ, Ezgi ERSOY, Mehmet BOĞA, Tuğçe BORAN, Ercan ÇINAR, Gül ÖZHAN, Ahmet Ceyhan GÖREN, Esra EROĞLU ÖZKAN

**Affiliations:** 1 Department of Pharmacognosy, Faculty of Pharmacy, İstanbul University, İstanbul Turkey; 2 Department of Pharmacognosy, Faculty of Pharmacy, Karadeniz Technical University, Trabzon Turkey; 3 Department of Analytical Chemistry, Faculty of Pharmacy, Dicle University, Diyarbakır Turkey; 4 Department of Pharmaceutical Toxicology, Faculty of Pharmacy, İstanbul University, İstanbul Turkey; 5 Department of Nursing, School of Health Sciences, Batman University, Batman Turkey; 6 Department of Analytical Chemistry, Faculty of Pharmacy, Bezmiâlem Vakıf University, İstanbul Turkey

**Keywords:** *Hypericum androsaemum*, cytotoxic, apoptosis, quillaic acid, antioxidant, LC-HRMS

## Abstract

The study aims to determine the secondary metabolites of *Hypericum androsaemum *L*. *extracts by liquid chromatography-high resolution mass spectrometry (LC-HRMS), and investigate the antioxidant and cytotoxic activities of the plant. Cytotoxic activity was evaluated by MTT assay, and apoptosis induction abilities on human prostate adenocarcinoma (PC-3), and hepatocellular carcinoma (Hep G2) cell lines. Accordingly, major secondary metabolites were found as hederagenin (762 ± 70.10 μg/g) in the leaves dichloromethane (LD), herniarin (167 ± 1.50 μg/g) in fruit dichloromethane (FD), (-)-epicatechin (6538 ± 235.36 μg/g) in the leaves methanol (LM), (-)-epigallocatechin gallate (758 ± 20.46 μg/g) in the fruit methanol (FM), and caffeic acid (370 ± 8.88 μg/g) in the fruit water (FW), and (3313 ± 79.51 μg/g) in the leaves water (LW) extracts. LM exerted strong antioxidant activity in DPPH free (IC_50_ 10.94 ± 0.08 μg/mL), and ABTS cation radicals scavenging (IC_50_ 9.09 ± 0.05 μg/mL) activities. FM exhibited cytotoxic activity with IC_50 _values of 73.23 ± 3.06 µg/mL and 31.64 ± 2.75 µg/mL on PC-3 and Hep G2 cell lines, respectively. Being the richest extract in terms of quillaic acid (630 ± 18.9 μg/g), which is a well-known cytotoxic triterpenoid with proven apoptosis induction ability on different cells, FM extract showed apoptosis induction activity with 64.75% on PC-3 cells at 50 μg/mL concentration. The study provides promising results about the potential of *Hypericum androsaemum* on cancer prevention.

## 1. Introduction

The genus *Hypericum* (Hypericaceae) is a large genus represented by nearly 500 species throughout the world. On the basis of the current knowledge, there are recently 109 taxa evaluated under 20 sections in Turkey, and 48 taxa of them are endemic [1]. The most popular member of the genus is undoubtedly *H. perforatum*, (common St. John’s wort), which has been used since ancient times mainly for its antidepressant and wound healing effects. Being one of the best-selling herbal medicines in the world, *H. perforatum* can be found in many stores in different forms of products, such as capsules, ointments, and drops. Incidentally, in the late nineties, preparations made with *H. perforatum* had made up as much as 25% of all prescriptions prescribed by doctors to treat depression in Germany [2].

In the past few years, there has been growing interest surrounding *Hypericum* species specifically, and as a consequence, the number of studies conducted to investigate their medical properties has increased exponentially. According to these studies, it can be said that the modern history of *Hypericum* species is beyond doubt a success story, and the reasons behind the success are rationale [3]. Being another member of the genus *Hypericum*, *Hypericum androsaemum* L. (common Tutsan) is an evergreen or semievergreen shrub that is widely known in the Mediterranean basin. It has been reported that the plant has been predominantly used for its sedative, antidepressant, anxiolytic, hepatoprotective, wound healing, antihypertensive, anti haemorrhoidal, and diuretic properties traditionally [4–7]. Differently from *H. perforatum*, not only the aerial parts but the fleshy and berry-like capsules that ripen from red to black produced by *H. androsaemum* have also been used for various medical purposes. Since the biological activities of *Hypericum* species are mainly attributed to their rich mixture of phytochemicals that includes naphthodianthrone derivatives (hypericin, pseudohypericin, etc.), acylphloroglucinols (hyperforin, adhyperforin, etc.), flavonoids (quercetin, hyperoside, etc.), tannins, xanthones, and volatile oils [8], it has become crucial to determine the chemical composition of *H. androsaemum* as well. As a corollary, there are several studies concerning the evaluation of the chemical composition and biological activities of *H. androsaemum*, including both *in vitro* and *in vivo* studies. It must be noted that *H. androsaemum* is a hypericin-free plant, which can be considered as a major advantage for especially skin-related activities since hypericin is a molecule that can cause phototoxicity, and can also induce activation of hepatic enzymes [9]. Interestingly, *H. androsaemum* is three times more expensive than *H. perforatum* in Portugal, due to the high demand and low supply compared to the famous *H. perforatum *[10].

Thus far, it has been reported by different studies that *H. androsaemum* is a quite rich plant in terms of phenolic compounds, including caffeoylquinic acids, chlorogenic acid, and quercetin derivatives [11,12]. With a better understanding of the chemical composition of the plant, it has been easier to more closely align the research designed to investigate the pharmacological properties. In this context, it has been reported by the studies published to date that *H. androsaemum* showed antioxidant [11], antidepressant [7,13], wound healing and UV protector [9], cytotoxic and immunomodulatory [6], acetylcholinesterase inhibitory [14], antiinflammatory, and anti-*Candida *[15] effects. 

Bearing all this in mind, this current study focuses on the secondary metabolite composition of *H. androsaemum* collected from Trabzon, Turkey by LC-HRMS analysis, and evaluation of its antioxidant and anticancer effects. Additionally, and most notably, as the search for safer and more effective agents against cancer, which is indisputably one of the most fatal diseases continues, this study provides a contribution to anticancer research with evidence for anticancer activity of the plant by showing cytotoxicity on prostate adenocarcinoma (PC-3), and hepatocellular carcinoma (Hep G2) cell lines, and significant apoptosis induction on PC-3 cell lines. As it has already been mentioned that both leaves and mature fruits were used traditionally for medical purposes, six extracts; dichloromethane, methanol, water extracts of leaves (LD, LM, LW), and mature fruits (FD, FM, FW) were prepared for the comparison of the activities. Lastly, this study aims to contribute new insights for understanding the therapeutic properties of *H. androsaemum.*

## 2. Material and methods

### 2.1. Chemical agents

**For LC-HRMS:** Caffeic acid (98%), emodin (90%), eupatilin (98%), astragalin (> 97%), (-)-epicatechin (≥ 90%), quercetin (≥ 95%), naringin (≥ 90%), rosmarinic acid (> 96%), naringenin (≥ 95%), chrysin (≥ 96%), fumaric acid (≥ 99%), and rutin (≥ 99%) were purchased from Sigma-Aldrich Germany. (+)-Catechin (> 97%,), (-)-epigallocatechin gallate (EGCG) (> 97%), epigallocatechin (> 97%), acacetin (> 97%), apigenin (> 97%), hederagenin (> 97%), quillaic acid (> 97), hyperoside (> 97%), 3-*O*-methylquercetin (> 97%), (+)-trans-taxifolin (> 97%), scutellarein (> 97%) were purchased from TRC Canada. Nepetin-7-glucoside (> 97%), dihydrokaempferol (> 97%), and rhamnocitrin (> 97%), were puchased from Phytolab. Luteolin-7-rutinoside (> 97%, Carbosynth Limited), verbascoside (86.31%, HWI Analytik GmbH), herniarin (> 98%, Carl Roth GmbH), hesperidin (> 98%, J&K Scientific Ltd GmbH), (-)-nepetin (98%, Supelco), apigenin 7-glucoside (> 97%, EDQM CS) were obtained from different companies.

**For antioxidant activity:** ABTS (2,2-Azinobis (3-ethylbenzothiazoline-6-sulfonic acid) diammonium salt, purity: 97.5%) and BHT (butylated hydroxytoluene, ≥ 99%) were purchased from Merck (Germany); DPPH (2,2-diphenyl-1-picrylhydrazyl, ≥ 95%), pyrocatechol (≥ 99%), CuCl_2_.2H_2_O (copper (II) chloride dihydrate, ≥ 99%), neocuproine (2,9-dimethyl-1,10-phenanthroline, ≥ 98%), EDTA (Ethylenediaminetetraacetic acid, ≥ 98%), and a-tocopherol (≥ 95.5%) were obtained from Sigma–Aldrich (Germany); Folin Ciocalteu phenol reagent was purchased from Applichem (Germany). All solvents were analytical grade.

**For cytotoxic activity and apoptotic determination:** MTT (3-(4,5-dimethylthiazol-2-yl)-2,5-diphenyltetrazolium bromide) was purchased from Biomatik (Cambridge, Ontario, Canada). Dimethylsulfoxide (DMSO) was obtained from Merck (Darmstadt, Germany). Sodium dodecyl sulfate (SDS) and all the cell culture materials were purchased from Wisent Bioproducts (Saint-Jean-Baptiste, QC, Canada). 

**Cell culture:** Prostate adenocarcinoma (PC-3) and hepatocellular carcinoma (Hep G2) cell lines were obtained from American Type Culture Collection (ATCC, VA, USA). The cells were grown in DMEM-F12 medium, following the manufacturer’s instructions. The subculture was performed when the cells reached 70-80% confluence every 2-3 days.

### 2.2. Plant material 

Leaves and mature fruits of *Hypericum* *androsaemum* L. were collected from Trabzon, Arsin, Çiçekli Village in August 2018. A voucher specimen was deposited in the Herbarium of Faculty of Pharmacy of İstanbul University (ISTE 116811).

### 2.3. Preparation of the extracts for bioactivity assays and LC-HRMS analysis

The air-dried plant material (leaves and mature fruits) was ﬁnely powdered, then separately weighed (10 g), and then macerated in methanol (100 mL) for 24 h at room temperature. The macerate was ﬁltered and this procedure was repeated three times. The ﬁltrates were gathered and then the methanol was evaporated under reduced pressure at below 45 °C. The same procedure was applied to obtain the dichloromethane extracts from the leaves and mature fruits. The water extracts of the leaves and mature fruits were prepared by using hot water infusion. The crude methanol extracts and infusions were lyophilized and stored at –20 °C.

### 2.4. LC-HRMS analysis

Thermo Orbitrap Q-Exactive instrument in ESI (Electrospray ionization) Source and Troyasil HS C18 column (150 mm × 2.1, 3 mm particle size) were used for LC-HRMS (Liquid Chromatography High-Resolution Mass Spectrometry) analysis. The gradient method (0–01.00 min 40% A and 60% B, 01.01–05.00 min 100% B, and finally 05.01–10.00 min 40% A and 60% B) was employed with 0.3 mL/min flow rate at 25 °C. The mobile phase A was formic acid:water (1:1000; v/v) and B is formic acid:methanol (1:1000; v/v). The injection volume was 2 μL. ESI source was utilized to obtain good ionization of small and relatively polar compounds. The scanning ion range was between m/z 85–1500 in the high-resolution mode of the instrument. The compound identification was performed by comparison of retention time of standard compounds (in the range of purity 95%–99%) and HRMS data of Bezmiâlem Vakıf University, Drug Application and Research Center Library (ILMER).

The extract was dissolved in the mobile phase (2.5 mL; A:B; 50:50; v/v), and then internal standard (100 mg/L; curcumin; 97%) was added to the final concentration of 3 ppm and volume was filled with mobile phase mixture up to 5 mL. Then, the solution was filtered through a 0.45 m filter and 2 mL was injected into the instrument [16–18].

### 2.5. Determination of total phenolic and flavonoid contents

UV spectrophotometer (Shimadzu) was used to determine total phenolic and flavonoid contents. 

#### 2.5.1. Determination of total phenolic contents

The method which was used to determine the total phenolic contents of the extract was described by Karapandzova et al. [19]. The phenolic compounds concentration was calculated using the following equation obtained from the standard pyrocatechol graphic, and the results were given as micrograms of pyrocatechol equivalents (PEs).

Absorbance = 0.0276 pyrocatechol (μg) + 0.0416 (R^2 ^ = 0.9987)

#### 2.5.2. Determination of total flavonoid content

Total flavonoid content determination of the extracts was evaluated according to Karapandzova et al. [19]. The flavonoid compounds concentration was computed using the equation (Absorbance = 0.0277 quercetin (μg) – 0.0434; R^2 ^ = 0.9992) obtained from standard quercetin graphic.

### 2.6. Antioxidant activity studies

A microplate reader (BioTek Power Wave XS, USA) was used for the antioxidant activity assays.

#### 2.6.1. DPPH radical scavenging activity

The method reported by Karapandzova et al. was applied to determine the DPPH free radical scavenging potential of the extracts [19]. The results were given as IC_50_ (The fifty percent inhibitory concentrations) values to express the antioxidant potential of the extracts.

#### 2.6.2. ABTS cation radical decolorization assay

The assay used to determine the scavenging capability of ABTS^•+^ in the extracts was detailedly defined by Li et al. [20]. IC_50_ values were calculated to indicate the scavenging capability of ABTS^•+^ in the extracts.

#### 2.6.3. Determination of metal chelating activity

The metal chelating potential of the extracts was tested using the method of Decker and Welch [21]. Chelating activity was calculated as IC_50 _values of the ferrozine – Fe^+2^ complex formation in the extracts.

#### 2.6.4. Cupric reducing antioxidant capacity (CUPRAC) method

The method described in detail by Apak et al. was carried out to identify the cupric reducing antioxidant capacity of the extracts [22]. The absorbance values of the extracts read at 450 nm. The results were given as A0.5 values with standard deviations and A0.5 values were calculated by absorbance-concentration graph. 

### 2.7. Cytotoxic activity studies

#### 2.7.1. MTT assay

The extracts were dissolved in DMSO or water depending on solubility in the concentration range of 12.5–100 µg/mL. The negative (untreated, culture medium) solvent (1% DMSO or water) and positive controls (0.1% SDS) were used in all tests. To calculate the 50% inhibition concentration (IC_50_), the absorbance values of each sample were compared with the absorbance values of the solvent controls. The IC_50_ value was computed as the percentage of solvent controls according to the following formula:

Inhibition (%) = 100 + [(corrected mean OD_sample_ × 100) / corrected mean OD_solvent control_]

The cell viability was determined through the mitochondrial pathway in the MTT assay described by Morgan [23]. The PC-3 cells were seeded in 96-well plates at 1 × 10^4 ^cells per well. The exposure time with the herbal extracts was 24 h. The exposure concentrations were in the range of 12.5–200 µg/mL in DMSO or water. After the cell treatments, 5 mg/mL MTT was added to 100 µL medium and the cells were incubated for 3 h at 37 °C in the dark. Following this step, the medium was moved away and the wells were rinsed with PBS. Then 100 µL of DMSO was added to each well and the plate was gently shaken for 10 min to dissolve the purple formazan crystals. Formazan product optical density (OD) was quantified at 590 nm against reference wavelength 670 nm using a microplate reader (Biotek, Epoc, VT, USA). All assays were independently repeated three times.

#### 2.7.2. Detection of apoptosis 

The detection of apoptosis was performed with fluorescein isothiocyanate (FITC) annexin V Apoptosis Detection Kit with propidium iodide (PI) (Biolegend, Koblenz, Germany) by ACEA Novo Cyte flow cytometer (San Diego, CA, USA). According to the manufacturer’s instructions, annexin V fluorescent dye binds to phosphatidylserine that is normally found in the inner part of the plasma membrane and externalizes apoptosis. PI directly binds to DNA. The cells are grouped (i) annexin V-negative and PI-negative (live cells), (ii) annexin V-positive and PI-negative (early apoptotic), and (iii) annexin V-positive and PI-positive (late apoptotic or necrotic) [24,25]. Briefly, the cells were seeded at 2 × 10^5^ cells/well in a 2 mL culture medium into a 6-well plate and incubated overnight. Then, the cells were treated with the highest concentrations of extracts for 24 h and 1% DMSO was used as a negative control. After trypsinization, the cells were collected and washed with cell staining buffer (Biolegend, Koblenz, Germany). After washing, the cells were resuspended with the density of 1 × 10^5^ cells/100 μL in annexin V binding buffer and 5 μL FITCH annexin V/10 μL PI were added, mixed, and incubated for 15 min at room temperature. After incubation, 400 μL annexin V binding buffer was added and fluorescence intensity was measured on an ACEA Novo Cyte flow cytometer (San Diego, CA, USA). The results were analyzed with NovoExpress software and data were expressed as the percent of the total cell amount.

### 2.8. Statistical analysis

All tests were repeated in triplicate. A t-test with Microsoft Excel was used to evaluate the results and the results were expressed as mean ± standard deviation. Differences were considered significant at p < 0.05.

## 3. Results and discussion

### 3.1. LC-HRMS analysis

In this study, chemical compositions of dichloromethane, methanol, water extracts of leaves (LD, LM, LW), and mature fruits (FD, FM, FW) of *H. androsaemum* collected from Arsin, Trabzon were investigated by LC-HRMS, and the results were given in Table 1. 

**Table 1:** Quantitative determination (μg /g) of 40 phytochemicals in the extracts of Hypericum androsaemum.

		Content of extracts (μg/g)					
Compounds	LD	FD	LM	FM	LW	FW	U %(k = 2)
Fumaric acid	<LOD	67 ± 2.14	16 ± 0.51	107 ± 3.42	<LOD	44 ± 1.40	3.2
Herniarin	150 ± 1.35	167 ± 1.50	144 ± 1.29	142 ± 1.27	123 ± 1.10	117 ± 1.05	0.9
Caffeic acid	<LOD	<LOD	134 ± 3.21	<LOD	3313 ± 79.51	370 ± 8.88	2.4
Chrysin	118 ± 1.41	<LOD	<LOD	<LOD	<LOD	<LOD	1.2
Apigenin	<LOD	<LOD	<LOD	<LOD	<LOD	<LOD	2.7
Emodin	<LOD	<LOD	<LOD	<LOD	<LOD	44 ± 1.05	2.4
Naringenin	82 ± 3.44	77 ± 3.23	86 ± 3.61	83 ± 3.48	88 ± 3.69	81 ± 3.40	4.2
Isosakuranetin	<LOD	<LOD	<LOD	<LOD	<LOD	<LOD	1.2
Acacetin	<LOD	<LOD	<LOD	<LOD	<LOD	<LOD	1.5
Luteolin	<LOD	<LOD	59 ± 1.12	<LOD	57 ± 1.08	<LOD	1.9
Kaempferol	120 ± 1.08	100 ± 0.90	896 ± 8.06	156 ± 1.40	175 ± 1.57	<LOD	0.9
Scutellarein	<LOD	<LOD	50 ± 0.7	<LOD	48 ± 0.67	<LOD	1.4
Dihydrokaempferol	<LOD	<LOD	<LOD	<LOD	3 ± 0.11	<LOD	3.8
(-)-Epicatechin	314 ± 11.30	<LOD	6538 ± 235.36	<LOD	882 ± 31.75	<LOD	3.6
(+)-Catechin	103 ± 1.85	<LOD	1122 ± 20.19	<LOD	172 ± 3.09	<LOD	1.8
Hispidulin	<LOD	<LOD	<LOD	<LOD	<LOD	<LOD	1.7
Rhamnocitrin	<LOD	<LOD	<LOD	<LOD	<LOD	<LOD	3.2
(+)-Trans taxifolin	129 ± 3.87	<LOD	171 ± 5.13	<LOD	303 ± 9.09	76 ± 2.28	3.0
Quercetin	137 ± 1.78	118 ± 1.53	804 ± 10.45	168 ± 2.18	183 ± 2.37	<LOD	1.3
(-)-Epigallocatechin	<LOD	<LOD	157 ± 4.86	<LOD	<LOD	<LOD	3.1
3-O-Methylquercetin	<LOD	<LOD	103 ± 3.09	<LOD	<LOD	<LOD	3.0
Nepetin	<LOD	<LOD	<LOD	<LOD	<LOD	<LOD	2.8
Penduletin	250 ± 4.00	<LOD	286 ± 4.57	336 ± 5.37	170 ± 2.72	<LOD	1.6
Eupatilin	228 ± 3.19	<LOD	266 ± 3.72	317 ± 4.43	146 ± 2.04	<LOD	1.4
Rosmarinic acid	<LOD	<LOD	65 ± 2.86	<LOD	62 ± 2.72	<LOD	4.4
Apigenin-7-glucoside CRS	<LOD	<LOD	<LOD	<LOD	<LOD	<LOD	3.1
Astragalin	<LOD	92 ± 3.86	130 ± 5.46	<LOD	<LOD	<LOD	4.2
Orientin	<LOD	81 ± 3.07	124 ± 4.71	<LOD	<LOD	<LOD	3.8
Quercitrin	<LOD	81 ± 3.88	124 ± 5.95	<LOD	<LOD	<LOD	4.8
(-)-Epigallocatechin gallate	193 ± 5.21	<LOD	3035 ± 81.94	758 ± 20.46	376 ± 10.15	141 ± 3.80	2.7
Myricitrin	198 ± 6.13	<LOD	3657 ± 113.36	<LOD	158 ± 4.89	<LOD	3.1
Hyperoside	229 ± 6.87	<LOD	3713 ± 111.39	<LOD	188 ± 5.64	<LOD	3.0
Hederagenin	762 ± 70.10	<LOD	<LOD	<LOD	<LOD	<LOD	9.2
Nepetin-7-glucoside	<LOD	<LOD	<LOD	<LOD	<LOD	<LOD	4.4
Quillaic acid	<LOD	<LOD	<LOD	630 ± 18.9	<LOD	298 ± 8.94	3.0
Naringin	<LOD	<LOD	<LOD	<LOD	<LOD	<LOD	2.8
Luteolin-7-rutinoside	<LOD	<LOD	<LOD	<LOD	<LOD	<LOD	1.4
Hesperidin	<LOD	<LOD	<LOD	<LOD	<LOD	<LOD	2.8
Rutin	139 ± 6.25	<LOD	232 ± 10.44	<LOD	66 ± 2.97	<LOD	4.5
Verbascoside	<LOD	<LOD	<LOD	<LOD	<LOD	<LOD	3.6

#### 3.1.1. Optimization of LC-HRMS

To avoid the similar MS/MS pattern of the triple quadrupole mass spectrometry [18,26], it was decided to use high-resolution mass spectrometry and MS/MS screening for the secondary metabolites of the extracts of *H. androsaemum*. In this technique, we have recorded the high-resolution mass spectrometry of each compound together with MS/MS ions simultaneously using Thermo Orbitrap Q Exactive instrument. Quantification of the compounds was carried out by the data of HRMS while for the identification of each metabolite HRMS, MS/MS and RT data of the standards were used. One of the most critical issues of the applied method is the dissolution of plant extracts in the appropriate solvent to get clear and repeatable separation of chromatographic peaks and ionization stability. Regarding previous reports of literature [18,26] and the findings clearly showed that one of the best mobile phase solution was determined to be a gradient of acidified methanol and water in the ESI source for those polar compounds.

#### 3.1.2. Method validation of LC-HRMS method

Validation of the applied method was performed using analytical standards of corresponding compounds (see subsection 2.1) using the target ions (Table 2) and curcumin was used as an internal standard.

**Table 2:** Analytical parameters of the LC–HRMS method for the phytochemicals.

Compound	Retention time	Ionization mode	Foundm/z	Calculatedm/z	Error(ppm)	Liner regresionequation	R²	LOD/LOQ*	Recovery %
Fumaric acid	2.46	Negative	115.0036	116.0104	3.15	y = 0.02396x + 0.00414	0.998	0.21/0.70	94.5
Herniarin	5.59	Positive	177.0546	176.0468	0.94	y = 5.23600 x + 0.72060	0.998	0.14/0.46	106.8
Caffeic acid	3.15	Negative	179.0340	180.0417	2.41	y = 0.64270x + 0.05448	0.998	0.12/0.41	106.5
Chrysin	6.97	Positive	255.0651	254.0574	1.19	y = 1.71000 + 0.22550	0.997	0.11/0.37	105.9
Apigenin	6.17	Negative	269.0455	270.0534	2.72	y = 0.74000 x + 1.17100	0.999	0.15/0.50	101.3
Emodin	8.40	Negative	269.0455	270.0534	2.39	y = 6.84500 x + 8.01000	0.999	0.10/0.35	104.0
Naringenin	5.72	Negative	271.061	272.0679	4.15	y = 0.81220 x +0.04423	0.998	0.10/0.33	96.4
Isosakuranetin	6.54	Negative	285.0768	286.0836	1.21	y = 1.36000 x + 0.15420	0.998	0.20/0.60	108.6
Acacetin	7.01	Positive	285.0757	284.0690	1.50	y = 0.60420 x + 0.64820	0.996	0.20/0.62	104.3
Luteolin	5.84	Positive	287.0550	286.0472	1.91	y = 0.15090 x + 0.03393	0.997	0.16/0.55	102.1
Kaempferol	6.05	Positive	287.0550	286.0472	0.91	y = 0.13150 x + 0.02696	0.997	0.15/0.51	102.9
Scutellarein	5.56	Positive	287.0550	286.0472	1.44	y = 0.11820 x + 0.00060	0.998	0.10/0.30	96.4
Dihydrokaempferol	4.84	Negative	287.0561	288.0628	3.80	y = 0.84890x + 0.14350	0.999	0.12/0.39	102.7
(-)-Epicatechin	2.54	Positive	291.0863	290.0785	3.62	y = 0.08876x + 0.02122	0.998	0.17/0.55	103.4
(+)-Catechin	2.28	Positive	291.0863	290.0785	1.84	y = 0.08329x + 0.00524	0.998	0.13/0.45	103.4
Hispidulin	6.20	Positive	301.0706	300.0628	1.73	y = 0.53940 x + 0.13180	0.999	0.10/0.30	107.8
Rhamnocitrin	6.94	Positive	301.0706	300.0628	3.21	y = 0.15590 x + 0.25980	0.999	0.14/0.46	108.4
(+)-Trans taxifolin	3.80	Negative	303.0510	304.0578	2.97	y = 0.24450x - 0.01517	0.998	0.12/0.44	102.5
Quercetin	5.66	Positive	303.0499	302.0421	1.30	y = 0.06643 x - 0.01097	0.984	0.33/1.10	108.1
(-)-Epigallocatechin	2.11	Positive	307.0812	306.0734	3.11	y = 0.04801x - 0.00370	0.998	0.16/0.56	104.7
3-O-Methylquercetin	6.10	Negative	315.0510	316.0578	2.95	y = 0.08915 x +0.341	0.997	0.23/0.77	112.4
Nepetin	5.86	Negative	315.0510	316.0578	2.76	y = 0.79200 x + 0.00364	0.998	0.10/0.33	92.9
Penduletin	6.58	Positive	345.0968	344.0891	1.58	y = 3.387 x + 1.2	0.999	0.06/0.20	101.65
Eupatilin	6.59	Positive	345.0968	302.0057	1.38	y = 3.178 x + 0.9574	0.997	0.20/0.66	106.9
Rosmarinic Acid	4.64	Negative	359.0772	360.0840	4.38	y = 0.09963x - 0.00258	0.999	0.06/0.20	100.8
Apigenin 7-glucoside	4.98	Positive	433.1129	432.1051	3.13	y = 0.09784x + 0.00958	0.997	0.11/0.37	107.9
Kaempferol-3-O-glucoside	4.20	Positive	449.1078	448.1000	4.15	y = 0.07406x + 0.01329	0.996	0.20/0.60	113.75
Orientin	3.09	Positive	449.1078	448.1000	3.83	y = 0.05944x - 0.00002	0.997	0.10/0.32	104.9
Quercitrin	5.12	Positive	449.1078	448.1000	4.78	y = 0.07564 x + 0.00577	0.99	0.12/0.38	116.4
(-)-Epigallocatechin gallate	2.10	Negative	457.0776	458.0844	2.65	y = 0.03253x + 0.00072	0.997	0.10/0.30	100.3
Myricitrin	4.56	Positive	465.1027	464.0949	3.11	y = 0.03812x + 0.00508	0.998	0.12/0.41	102.4
Hyperoside	4.56	Positive	465.1027	464.0949	3.01	y = 0.03837x + 0.00357	0.998	0.11/0.40	103.7
Hederagenin	8.56	Negative	471.3479	472.3547	9.23	y = 0.0031x + 0.009	0.994	0.90/2.90	96.5
Nepetin-7-glucoside	4.55	Negative	477.1038	478.1106	4.39	y = 0.11530x + 0.00497	0.999	0.06/0.20	103.6
Quillaic acid	7.41	Negative	485.3272	486.3340	3.01	y = 0.29780 x + 0.01883	0.999	0.08/0.26	102.8
Naringin	4.08	Negative	579.1719	580.1787	2.84	y = 0.05422x - 0.00255	0.999	0.10/0.30	105.9
Luteolin-7-rutinoside	3.82	Negative	593.1511	594.1579	1.43	y = 0.07102x - 0.01863	0.998	0.20/0.60	99.5
Hesperidin	4.36	Negative	609.1824	610.1892	2.82	y = 0.03076x - 0.00253	0.999	0.11/0.36	105.7
Rutin	4.46	Negative	609.1461	610.1528	4.47	y = 0.03758x + 0.00045	0.999	0.10/0.31	103.3
Verbascoside	2.87	Negative	623.1981	624.2049	3.59	y = 0.05182x + 0.00063	0.998	0.13/0.41	108.7

Parameters of equipment: Thermo Orbitrap Q-Exactive instrument in ESI Source and Troyasil HS C18 column (150 mm × 2.1, 3 mm particle size) were used for LC-HRMS analysis. The gradient method (0–01.00 min 40% A and 60% B, 01.01–05.00 min 100 % B, and finally 05.01–10.00 min 40% A and 60% B) was employed with 0.3 mL/min flow rate at 25 °C. The mobile phase A was formic acid:water (1 : 1000; v / v) and B is formic acid:methanol (1:1000; v/v). The injection volume was 2 μL.

##### 3.1.2.1. Linearity

Calibration curves were obtained from standard calibration solutions. The linearity of the method was assayed by analyzing the calculation of a six-point linear plot in the range of 0.1 mg/L to 10 mg/L with six replicates. The linear regression equation and the squared correlation coefficients were determined and reported in Table 2. 

##### 3.1.2.2. LOD and LOQ

The limit of detection (LODs) of the method for each compound was determined according to the following equation: LOD or LOQ (The limit of quantification) = κSDa/b, where 3 for LOQ and κ = 3 for LOD, SDa represents the standard deviation of the intercept, and b represents the slope (Table 2) [18, 26–28].

##### 3.1.2.3. Recovery, repeatability, and intermediate precision

Repeatability, and intermediate precision of the developed LC-HRMS used and amount of secondary metabolites were measured in six different extracts for preliminary screening. Then, according to the detected levels of compounds in the extracts, we spiked the extracts to reach the final concentrations as 0.1 mg/L and 0.5 mg/L, and 1 mg/L in 5 mL volumetric flasks. Unspiked plant extracts were also analyzed to determine the target compound concentrations in the blank sample. The recovery of each component at each fortification level was calculated according to the following formula [26,28].

(1)Recovery %=Measured conncentration - endogeneous concentrationspiked concentrationx100

The recoveries of measured secondary metabolites were ranged from 94.5 to 116.4% (Table 2). Mean relative standard deviations (RSD) were found to be 1%–10 % for all and those data were added to the uncertainty budget of all reported compounds. 

 The repeatability of the applied method was assessed at three concentration levels of the recovery studies. For the intermediate (reproducibility) precision, a set of spiked samples having three concentration levels were analyzed twice a week for a period of 3 weeks and the repeatability of the method evaluated.

#### 3.1.3. Estimation of uncertainty

##### 3.1.3.1. Identification of uncertainty sources

The same uncertainty evaluation method, the bottom-up approach was applied to obtain the measurement of the uncertainty value for the developed method. The sources for uncertainty were determined as were weighing the sample, calibration graph, and repeatability. Detailed evaluation procedure corresponding equations are given in the previous papers [27,29,30] and to avoid repetition, we summarized the calculation of combined standard measurement uncertainty of target compounds in plant extracts in equation 2. The expanded measurement uncertainty was obtained by multiplying the combined standard measurement uncertainty value with 2 (coverage factor) at a 95% confidence level. The uncertainty value of measurement results is given in Table 2.

(2)uc(A)CA=(u(Wss)Wss)2+(u(C0)C0)2+u(Rm)2+u(r)2

where 

*u* *_c_* *(A):* Combined standard measurement uncertainty of the analyte, 

*C* *_A_*: Concentration of the target analyte,

*u(W* *_SS_* *)*: Combined standard measurement uncertainty of the sample intake,

*W* *_SS_*: Weight of the starting sample, 

*u(c* *_0_* *)*: Combined standard measurement uncertainty of the calibration curve, 

*c* *_0_*: Determined concentration of the sample by using the calibration curve, 

*u(R* *_m_* *)*: Combined standard measurement uncertainty of recovery, 

*u(r)*: Standard measurement uncertainty of repeatability.

#### 3.1.4. Identification of secondary metabolites

Two metabolites, naringenin, and herniarin were identified in all analyzed extracts.

LD extract had the highest total flavonoid content among the six studied extracts. As regards the LD extract, 15 constituents were identified. The most abundant secondary metabolite in the LD was hederagenin (762 ± 70.10 μg/g extract) which is a triterpenoid. Incidentally, hederagenin was only found in this one among the 6 studied extracts. Chrysin is another constituent that was determined only in LD with 118 ± 1.41 μg/g extract. Other compounds that were detected in LD were herniarin (150 ± 1.35 μg/g extract), naringenin (82 ± 3.44 μg/g extract), kaempferol (120 ± 1.08 μg/g extract), (-)-epicatechin (314 ± 11.30 μg/g extract), (+)-catechin (103 ± 1.85 μg/g extract), (+)-trans taxifolin (129 ± 3.87 μg/g extract), quercetin (137 ± 1.78 μg/g extract), penduletin (250 ± 4.00 μg/g extract), eupatilin (228 ± 3.19 μg/g extract), (-)-epigallocatechin gallate (193 ± 5.21 μg/g extract), myricitrin (198 ± 6.13 μg/g extract), hyperoside (229 ± 6.87 μg/g extract), and rutin (139 ± 6.25 μg/g extract). 

In the FD extract, 8 compounds were present. FD is the richest extract among the analyzed extracts in terms of a coumarin derivative, herniarin (167 ± 1.50 μg/g extract). Fumaric acid (67 ± 2.14 μg/g extract), naringenin (77 ± 3.23 μg/g extract), kaempferol (100 ± 0.90 μg/g extract), quercetin (118 ± 1.53 μg/g extract), astragalin (92 ± 3.86 μg/g extract), orientin (81 ± 3.07 μg/g extract), and quercitrin (81 ± 3.88 μg/g extract) are the other phytochemicals that were found in FD extract. 

A total of 23 different constituents were identified in the LM extract of *H. androsaemum*. It must be also noted that, among the six extracts that were investigated, LM had the highest total phenolic content. (-)-Epigallocatechin (157 ± 4.86 μg/g extract), and 3-*O*-methylquercetin (103 ± 3.09 μg/g extract) are the phytochemicals that were found only in LM. Additionally, LM was found to be richer than the other analyzed extracts in terms of luteolin (59 ± 1.12 μg/g extract), kaempferol (896 ± 8.06 μg/g extract), scutellarein (50 ± 0.70 μg/g extract), (-)-epicatechin (6538 ± 235.36 μg/g extract), (+)-catechin (1122 ± 20.19 μg/g extract), quercetin (804 ± 10.45 μg/g extract), rosmarinic acid (65 ± 2.86 μg/g extract), astragalin (130 ± 5.46 μg/g extract), orientin (124 ± 4.71 μg/g extract), quercitrin (124 ± 5.95 μg/g extract), (-)-epigallocatechin gallate (3035 ± 81.94 μg/g extract), myricitrin (3657 ± 113.36 μg/g extract), hyperoside (3713 ± 111.39 μg/g extract), and rutin (232 ± 10.44 μg/g extract). Besides, fumaric acid (16 ± 0.51 μg/g extract), herniarin (144 ± 1.29 μg/g extract), caffeic acid (134 ± 3.21 μg/g extract), naringenin (86 ± 3.61 μg/g extract), (+)-trans taxifolin (171 ± 5.13 μg/g extract), penduletin (286 ± 4.57 μg/g extract), and eupatilin (266 ± 3.72 μg/g extract) were the other metabolites that were present in LM. 

In FM, 9 compounds were detected. In terms of fumaric acid (107 ± 3.42 μg/g extract), penduletin (336 ± 5.37 μg/g extract), eupatilin (317 ± 4.43 μg/g extract), and quillaic acid (630 ± 18.9 μg/g extract), FM extract was found to be the richest. Also, herniarin (142 ± 1.27 μg/g extract), naringenin (83 ± 3.48 μg/g extract), kaempferol (156 ± 1.40 μg/g extract), and (-)-epigallocatechin gallate (758 ± 20.46 μg/g extract) were identified in the extract. 

In the FW extract of *H. androsaemum*, 8 metabolites were determined. An anthraquinone derivative emodin was found only in this extract with 44 ± 1.05 μg/g extract. Fumaric acid (44 ± 1.40 μg/g extract), herniarin (117 ± 1.05 μg/g extract), caffeic acid (370 ± 8.88 μg/g extract), naringenin (81 ± 3.40 μg/g extract), (+)-trans taxifolin (76 ± 2.28 μg/g extract), (-)-epigallocatechin gallate (141 ± 3.80 μg/g extract), and quillaic acid (298 ± 8.94 μg/g extract) are the other constituents of the FW extract according to the results. 

LW is also found to be quite a rich extract with 18 metabolites in it. The major compound of the extract was caffeic acid with 3313 ± 79.51 μg/g extract. Also, in terms of (+)-trans taxifolin (303 ± 9.09 μg/g extract), LW was more abundant than the other extracts. Herniarin (123 ± 1.10 μg/g extract), naringenin (88 ± 3.69 μg/g extract), luteolin (57 ± 1.08 μg/g extract), kaempferol (175 ± 1.57 μg/g extract), scutellarein (48 ± 0.67 μg/g extract), dihydrokaempferol (3 ± 0.11 μg/g extract), (-)-epicatechin (882 ± 31.75 μg/g extract), (+)-catechin (172 ± 3.09 μg/g extract), quercetin (183 ± 2.37 μg/g extract), penduletin (170 ± 2.72 μg/g extract), eupatilin (146 ± 2.04 μg/g extract), rosmarinic acid (62 ± 2.72 μg/g extract), (-)-epigallocatechin gallate (376 ± 10.15 μg/g extract), myricitrin (158 ± 4.89 μg/g extract), hyperoside (188 ± 5.64 μg/g extract), and rutin (66 ± 2.97 μg/g extract) were found to be present in LW extract.

A few number of studies have been carried on the investigations on chemical constituents of *H. androsaemum *growing in Turkey. In a previous study, ethanol extract of aerial parts of the plant was obtained and the chemical composition of the extract was analyzed using an LC/ESI/MS/MS method. According to the results of the study, rutin, hyperoside, quercitrin, and quercetin were identified in the extracts [31]. Another study was also conducted in 2017 to determine the secondary metabolites of *H. androsaemum* that were collected from different altitudes of Turkey by HPLC. The results showed that chlorogenic acid, neochlorogenic acid, caffeic acid, hyperoside, isoquercitrin, quercitrin, quercetin, (+)-catechin, and (-)-epicatechin were identified in the whole plant material of *H. androsaemum* sampled at different altitudes of a valley in Turkey [32].

As mentioned in the introduction part before, *H. androsaemum* is one of the most popular members of the Hypericaceae family, and not only distributed in Turkey but also widely and wildly grows in many parts of Europe. On that account, there have been studies focused on the phytochemical composition of the plant that was harvested from different countries as well. One of those studies that was conducted in Portugal showed that caffeoylquinic acid and quercetin derivatives were found to be the major compounds in the water infusion of the plant [11]. In another study, the HPLC/DAD analysis of methanol extracts of *H. androsaemum* harvested from Portugal revealed a phenolic fingerprint composed of different compounds including mainly caffeoylquinic acid, quercetin derivatives, and kaempferol [33]. The phytochemical profiles of ethanol extracts of different *Hypericum* species from Sicily-Italy were screened by HPLC-DAD-MS in 2018, and the results showed that the *H. androsaemum* ethanol extract contained caffeoylquinic acid, catechin, and a few quercetin derivatives [34]. According to another study, quinic acid, chlorogenic acid, and quercetin derivatives were identified in methanol extracts of aerial parts of the plant from Portugal [35]. According to the study about the chemical characterization of the ethanol extract from red berry-like fruits of *H. androsaemum*, the extract was found to be rich in terms of shikimic acid and chlorogenic acid. Moreover, neochlorogenic acid, gallic acid, catechin, epicatechin, rutin, and hyperoside were found to be present in the extract. This study was influential, regarding this was the first report about the marker bioactive compounds of *H. androsaemum* red berries, and more importantly, the significant phytochemicals that exhibit a wide range of biological activities such as chlorogenic acid was found to be much more abundant compared to the leaves extracts [7]. The chromatographic profile of the *H. androsaemum* ethanol:water extract was also reported by a previous study. Accordingly, protocatechuic acid, 3-*O*-caffeoylquinic acid, 5-*O*-caffeoylquinic acid, epicatechin, taxifolin, quercetin-3-*O*-glucuronide, and quercetin-3-*O-*glucoside were identified in the extract. The extracts also exerted antioxidant, cytotoxic, antiinflammatory, and anti-*Candida* activities, which were attributed to the major phenolic constituents, namely 3-*O*-caffeoylquinic acid and 5-*O*-caffeoylquinic acid [15]. Along with these studies, there have been few other studies to investigate the constituents of the extracts prepared with the berries of the plants. Shikimic acid, gallic acid, chlorogenic acid, catechin, epicatechin, rutin, and hyperoside were determined predominantly [6,7,9,13].

Within this context, the results of this study are in agreement with the literature, moreover, it can be seen that many of the phytochemical compounds were determined for the first time within this current analysis. 

### 3.2. Total phenolic and flavonoid contents

The concentrations of total phenolic and flavonoid of water, methanol, and dichloromethane extracts prepared from leaves, and fruits of *H. androsaemum* were given as micrograms of pyrocatechol (PE) and quercetin (QE) equivalents, respectively. The total phenolic content of the extracts was higher than the total flavonoid content except for LD. LM had the highest total phenolic content with 108.51 ± 1.28 μg pyrocatechol equivalent/mg extract, and LD had the highest total flavonoid content with 86.74 ± 2.29 μg quercetin equivalent/mg extract. The total phenolic and flavonoid contents values are given in Table 3. Generally, the total phenolic and flavonoid contents of leaf extracts are found to be higher than the fruit extracts. In the study of Keskin et al., total phenolic contents of flower, fruits, and seed methanol extracts of *H. retusum* were investigated. The total phenolic content of fruit methanol extract of *H. retusum *was found to be lower than the other extract [36]. In the study of Sekeroglu et al., ethanol extract of the leaves of *H. perforatum *had the highest total phenolic content among the flower, stem, and leaf extracts [37]. Another study indicated that methanol extract of *H. lydium* had higher total phenolic content than the water extract [38]. In the study of Saddiqe et al., the total flavonoid content of the dichloromethane extract of *H. androsaemum* was found to be higher than its total phenolic content [39]. According to the literature survey, total phenolic and flavonoid content results in this study are parallel with the other studies about total phenolic and flavonoid contents of different *Hypericum* species.

**Table 3:** Total phenolic and flavonoid content, and antioxidant activities of H. androsaemum extracts.

			IC50 values (mg/mL)			A0.5 (µg/mL)
Samples	Phenolic content(μg PEs/mg extract)†	Flavonoid content(μg QEs/mg extract)‡	DPPH free radical	ABTS cation radical	Metal chelate	CUPRAC
LD	46.01 ± 0.56	86.74 ± 2.29	67.58 ± 0.24	26.50 ± 0.62	141.06 ± 0.72	41.27 ± 0.55
FD	47.83 ± 1.56	9.24 ± 0.72	93.23 ± 1.06	68.81 ± 1.02	117.24 ± 1.55	37.68 ± 0.27
LM	108.51 ± 1.28	38.48 ± 0.51	10.94 ± 0.08	9.09 ± 0.05	96.56 ± 1.29	7.21 ± 0.05
FM	46.21 ± 0.26	8.28 ± 0.55	90.51 ± 0.86	48.22 ± 2.14	>1000	70.03 ± 0.91
LW	88.59 ± 1.28	31.02 ± 0.75	29.83 ± 1.06	10.88 ± 0.44	>1000	17.49 ± 0.58
FW	11.59 ± 0.00	2.14 ± 0.02	>1000	>1000	77.61 ± 0.79	81.28 ± 0.51
BHA	-	-	7.88 ± 0.20	2.74 ± 0.03	-	0.63 ± 0.02
α-TOC	-	-	16.30 ± 0.79	10.20 ± 0.05	-	13.38 ± 0.07
BHT	-	-	58.86 ± 0.50	3.16 ± 0.06	-	2.02 ± 0.01
EDTA	-	-	-	-	44.65 ± 0.43	-

*Values are means ± SD of three paralel measurements. † PEs, pyrocatechol equivalents y = 0.0276x + 0.0416 R2 = 0.9987.‡ QEs, quercetin equivalents y = 0.0277x + 0.0434 R2 = 0.9992.

### 3.3. Antioxidant activities

DPPH free radical scavenging, ABTS cation radical scavenging, and metal chelating activities results given as IC_50_ (concentration of 50% inhibition) and CUPRAC results given as A_0.5_ values (Table 3). 

In DPPH free radical scavenging activity, LM showed the best activity among all extracts and also from the standards a-TOC and BHT, with IC_50_ 10.94 mg/mL. LW also showed strong DPPH free radical scavenging activity, even stronger than BHT with IC_50_ 29.83 mg/mL. Keskin et al. reported DPPH free radical scavenging activities of flower, fruit and, seed extracts of *H. retusum*. Fruit extract showed weaker activity than the other extracts [36]. In the results of the current study, all fruit extracts of *H. androsaemum* showed weaker activity than all leaves extracts. Boga et al. reported methanol extract of *H. capitatum* var. *capitatum* had the highest DPPH free radical scavenging activity among the petroleum ether, acetone, methanol, and water extracts with IC_50_ 16.82 mg/mL [40]. DPPH free radical activities of ethanol and water extracts of flower, stem, and leaf of *H. perforatum* were studied, and all ethanol extracts showed better activities than water extracts. The leaf ethanol extract also showed the best activity with 84.0% inhibition value at 1 mg/mL concentration [37]. The results obtained from the current study showed that there was parallelism with literature results.

LW and LM showed strong antioxidant activity in ABTS cation radical scavenging assays with IC_50_ 10.88 ± 0.44 and 9.09 ± 05 mg/mL values, respectively, and methanol extract showed better activity than a-TOC which was used as standard. Methanol extract of *H. humifusum* with 180.87 mg TEs/g for ABTS exerted strong activity [41]. In the study of Eruygur et al., in ABTS cation radical scavenging assay, the methanol extract showed better activity (IC_50_ 16.63 μg/mL) than the water extract (IC_50_ 20.48 μg/mL) [38]. In ABTS cation radical scavenging assay, the methanol and water extracts of the whole *H. capitatum *var. *capitatum* showed very well activities with IC_50_ 9.24 and 9.76 μg/mL values, respectively. These values are almost identical to the result of the previous study conducted by Boga et al. (2016) [40]. In the study of Kang et al., ABTS radical scavenging activity of petroleum ether, ethyl acetate, and methanol extracts of the whole plants of *H. ascyron* were studied and methanol extract showed the best activity among all extracts with IC_50_ 24.55 μg/mL value [42]. There is only some literature about ABTS cation radical scavenging activity on *Hypericum* species and results are very similar to the current study results. 

FW demonstrated the best metal chelating activity among all extracts with IC_50_ 77.61 ± 0.79 mg/mL value. LM showed moderate metal chelating activity (IC_50_ 96.56 ± 1.29 mg/mL). In the study of Maltas et al., methanol extracts of *H. salsugineum*, *H. aviculariifolium,* and *H. perforatum* showed moderate metal chelating activities [43].

LM demonstrated significant cupric reducing antioxidant capacity (CUPRAC) and showed better activity than a-TOC that was used as standard at all tested concentrations. LW was also found to have good cupric reducing antioxidant capacity. In different studies, water extracts of *H. lanuginosum*, *H. capitatum* var. *capitatum *showed better activity than the other extract unlike our results [40,44]. Furthermore, the study of Ersoy et al. showed that the methanol extracts of three different *Hypericum* species especially *H. calycinum* have good cupric ion reducing antioxidant capacity [45].

### 3.4. Determination of cytotoxic activity

In the current study, cytotoxicity of FM, FD, FW, LM, LD, and LW extracts of *H. androsaemum* was measured on two cancer cell lines, namely prostate adenocarcinoma (PC-3) and hepatocellular carcinoma (Hep G2) cell lines. 0.1% SDS was used as the positive control. The results are given in Table 4, as the percentage of dead cells, and also the calculated half-maximal inhibitory concentration value (IC_50_) and comparing them by statistical analysis.

**Table 4:** The cytotoxic effects of H. androsaemum extracts on the PC-3 and Hep G2 cell lines (Inhibition (%) and IC50 values).

	PC-3 cell line						Hep G2 cell line					
Concentration(µg/mL)	LD	FD	LM	FM	LW	FW	LD	FD	LM	FM	LW	FW
200	N.S.	N.S.	29.34 ± 1.84	N.S.	N.S.	N.S.	N.S.	N.S.	34.57 ± 1.67	N.S.	N.S.	N.S.
100	N.S.	41.35 ± 3.02	13.30 ± 3.33	67.08 ± 1.02	3.34 ± 3.80	N.S.	N.S.	68.05 ± 1.38	14.09 ± 0.42	86.06 ± 0.35	14.64 ± 1.03	N.S.
50	28.33 ± 2.40	36.81 ± 3.27	15.84 ± 3.95	31.57 ± 0.54	9.06 ± 3.72	15.73 ± 3.52	44.12 ± 1.27	15.78 ± 0.22	4.79 ± 3.13	69.39 ± 0.52	5.42 ± 2.53	5.45 ± 2.63
25	14.31 ± 3.81	25.31 ± 1.96	16.81 ± 3.55	27.87 ± 0.84	18.20 ± 1.47	8.52 ± 1.90	10.94 ± 1.84	12.69 ± 0.07	3.53 ± 3.16	45.95 ± 2.02	1.87 ± 0.30	4.35 ± 2.24
12.5	4.85 ± 3.73	7.52 ± 3.39	11.28 ± 3.52	9.42 ± 2.05	17.64 ± 3.70	5.10 ± 1.14	8.59 ± 1.39	0.57 ± 1.06	1.03 ± 0.30	12.03 ± 1.72	2.66 ± 0.63	3.09 ± 1.86
IC50	>50	>100	>200	73.23 ± 3.06	>100	>50	>50	81.18 ± 0.45	>200	31.64 ± 2.75	>100	>50

Data were expressed as mean ± standard errors of the means (SEM). p < 0.05. N.S.: not studied concentration (The concentrations could not teste due to solubility problems for the extracts).

As known, a higher cytotoxic effect is indicated by a lower IC_50_ value. According to the results, FM was the most effective cytotoxic among the six studied extracts on both cell lines with IC_50 _values of 73.23 µg/mL on PC-3 cell lines, and 31.64 µg/mL on Hep G2 cell lines. FD exhibited weak cytotoxic activity on Hep G2 cell lines (IC_50_ 81.18 µg/mL). It must be noted that due to the solubility problems it has not been possible to test the cytotoxicity of these extracts with higher concentrations. The cytotoxic potential of the extracts may increase with prolonged exposure.

A remarkable fact that, out of 121 prescription drugs that were being used for cancer treatment, 90 of them are known as plant-based drugs [46]. Plant extracts that are rich in phenolic compounds, especially in flavonoids, have been proven to be strong candidates in the treatment of cancer, and they are considered as new horizons for cancer therapeutics. The conclusion reached by a variety of scientists is, there is a positive correlation between flavonoid intake and reduced incidence of cancer. For instance, they were found to be effective in the treatment of prostate cancer, which is one of the major medical burdens, being the most common cancer in males in Europe and North America, via modulating the apoptotic pathway [47,48]. On that account, flavonoid-polyphenol anticancer agents including *H. androsaemum* are becoming more intriguing with low or no toxicity and improved efficacy in the treatment of the disease.

**Figure 1:**
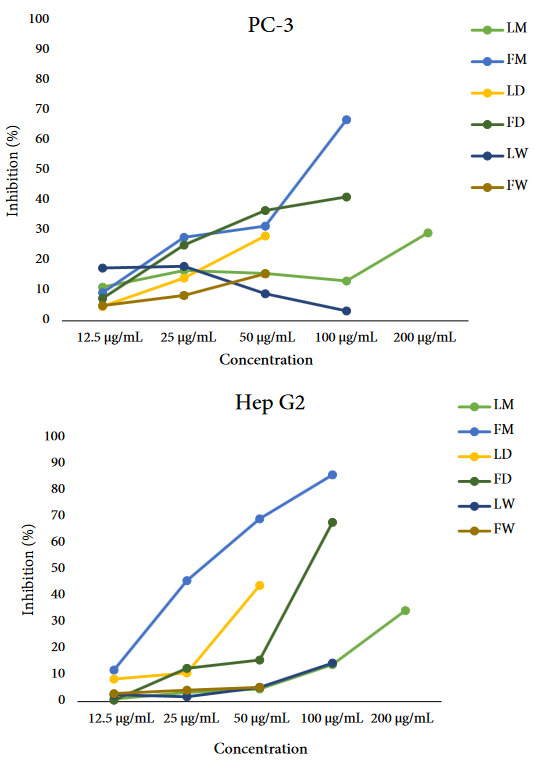


In the past years, a relatively large number of studies were devoted to different aspects of different biological activities, especially the cytotoxicity of triterpenoids. Therefore, there are numerous studies about the cytotoxicity of triterpenoids with promising results. Notwithstanding the fact that most studies were carried out on cell lines from one to five, which can be considered as a narrow range of cell lines, some studies investigated their cytotoxic activity on a wide panel of them, even up to 59 cell lines and they provided evidence for their cytotoxicity [49]. As mentioned above, the obtained results of this study suggest that the FM extract exhibited higher cytotoxic activity than the other extracts (Figure 1), and this significant difference in activity seems to be due to the presence of a certain compound, which is quillaic acid, a triterpenoid. Therefore, the results indicate that the anticancer activity of the extract could be related to quillaic acid.

On the other hand, it can be said that the understanding of anticancer properties with *Hypericum *species is substantially increased because there are studies that give information about investigations on cytotoxic activities of several *Hypericum* species. For instance, it is known that *H. pamphylicum* showed no cytotoxic activity against human cervical cancer (HeLa and NRK-52E) cell lines [50]. In another study, the cytotoxic effect against A549 and PC3 human lung cancer cell lines demonstrated by *H. adenotrichum* and *H. olympicum* was shown using the Comet assay. It was also reported that *H. adenotrichum* exhibited stronger cytotoxic activity than *H. olympicum* in a dose-dependent manner [51]. *H. japonicum* was also found to have cytotoxic effects, the methanol extract of the plant caused cytotoxicity on human acute monocytic leukemia (THP-1) cell lines [52]. There is another study about the cytotoxicity of *H. olympicum* and it has also given evidence about its cytotoxic activity on MCF-7 and MDA-MB-231 breast cancer cell lines [53]. Moreover, *H. triquetrifolium* methanol extract was shown to have a cytotoxic effect on different cell types including large cell lung carcinoma (COR-L23), hepatocellular carcinoma (Hep G2), renal adenocarcinoma (ACHN), amelanotic melanoma C32 cell lines [54]. *H. scabrum* is also among *Hypericum *species with cytotoxic activity on HT-29 and Hep G2 cell lines [55]. *H. androsaemum* has also been evaluated for its cytotoxic properties and reportedly, the red and black berry infusions demonstrated cytotoxic effect on colon carcinoma cells, such as HCT15 and CO115 cell lines [6]. In another study, *H. androsaemum* ethanol extract was found to be the most effective cytotoxic among 16 different *Hypericum* species on human glioblastoma A1235 cell lines [56]. Furthermore, a strong cytotoxic effect on NIH/3T3 murine fibroblasts was revealed by a study conducted in 2018 [34]. Over and above that, it was found to exhibit a cytotoxic effect on HeLa, Hep G2, MCF-7 (breast adenocarcinoma), and NCI-H460 (nonsmall cell lung cancer) cell lines [15]. To sum up, it would be accurate to say that the results of this study are in accordance with previous studies.

### 3.5. Determination of apoptotic potential

Apoptosis is a term that was first used in 1972 by scientists to describe a mode of cell death regulated by endogenously driven mechanisms, and a characteristic morphology [57]. One of the most important advances in basic cancer research was the discovery that apoptosis has a profound effect on the malignant phenotype, and it has been reported that most cytotoxic agents induce apoptosis [58].

Prostate cancer is the fifth most common cause of cancer death, and the most commonly diagnosed cancer in men globally, which is interestingly more common in the developed countries [48]. In the search of new natural products as agents against this disease, being traditionally used against prostate problems and quite rich in terms of flavonoids, *H. androsaemum* has been one of the candidates. Liver cancer is another most frequently occurring cancer in the world, being the second most common cause of cancer mortality [59]. It has been shown by the previous studies that *H. androsaemum* extracts induce apoptosis in human colorectal cells [60]. Human lung carcinoma A549, melanoma SK-Mel-2, and mouse skin melanoma B16F1 cells [61]. 

**Figure 2:**
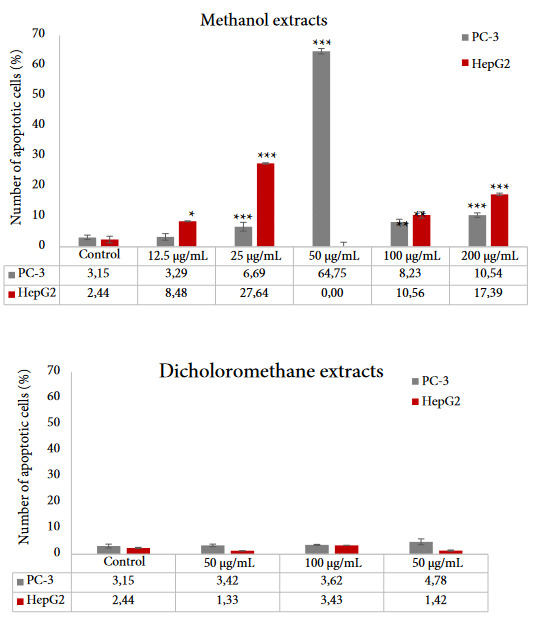


In this current study, the apoptosis assay was performed at different concentrations in the range of 12.5–200 µg/mL for the extracts. Treatment of 12.5, 25, and 50 µg/mL doses of FM on PC-3 cell lines, total apoptotic cells were detected as 3.29%, 6.69%, and 64.75%, respectively. Following the application of 12.5 and 25 μg/mL doses of FM on Hep G2 cells, 8.48% and 27.64% total apoptotic cells were observed, respectively. When 100 and 200 μg/mL of LM were applied on PC-3 cells, the apoptotic cell death was detected as 8.23% and 10.54%, respectively. 10.56% and 17.39% total apoptotic cells were determined after the application of 100 and 200 μg/mL of LM on Hep G2 cells, respectively. On the other hand, the dichloromethane extracts did not induce apoptosis on both cell lines. Considering the results of the Annexin V assay (Figure 2), FM extract showed remarkable selective apoptotic induction on PC-3 cells at 50 μg/mL concentration with nearly 65%.

Evaluating the chemical compounds of the FM extract compared to other studied extracts, it may be observed that the FM was the richest in terms of quillaic acid (630 ± 18.9 μg/g extract). As mentioned before, quillaic acid is a triterpenoid, and there is evidence about its apoptosis induction potential. In particular, in a study that was carried out to investigate its anticancer potential, it has been reported that quillaic acid was effective on the induction of apoptosis in human colon cancer cells [62], human Jurkat T lymphocytes [63], and gastric cancer AGS cells [64]. Furthermore, in 2005, it was reported that quillaic acid glycosides were shown to induce many apoptotic manifestations in cancer cells and therefore cancer-related activities are among the most popular properties of triterpenoids in the last decade [65]. Another major compound of the FM extract is (-)-epigallocatechin gallate, which is also proven to induce apoptosis [66]. However, EGCG could also act as an antiapoptotic agent in higher concentrations [67]. This can explain the lesser apoptotic activity of the LM extract, which stood out as the richest in terms of EGCG (3035 ± 81.94 μg/g extract). 

There are studies conducted by different scientists from all over the world investigating the apoptotic induction activity of various *Hypericum* species on different cell lines. For example, *H. ascyron* demonstrated apoptotic induction on HeLa cells [68]. *H. japonicum* ethyl acetate extract induced apoptosis on mouse H22 liver cancer cell lines in vivo [69]. *H. adenotrichum* and *H. olympicum* methanol extracts also induced apoptosis on human PC3 lung cancer cells [56]. Additionally, *H. scabrum* methanol extract and petroleum ether fraction were shown to reveal apoptosis induction capacity [55]. More importantly, *H. androsaemum* was also found effective in apoptosis induction in previous studies. It has been reported that aqueous extract of the leaves induced apoptosis on HCT115 and CO115 human colon carcinoma cell lines through MAPkinase and PI3K/Akt pathway [6,65].

Based on the results of this present study, which is the first report about the determination of quillaic acid in *H. androsaemum* to the best knowledge, and its apoptotic induction ability on Hep G2 and PC-3 cell lines, therefore it can be said that this could provide a new horizon for cancer therapeutics.

## 4. Conclusion

*H. androsaemum* is one of the most popular members of *Hypericum* species. A plethora of studies, most of which focus on the other pharmacological properties of them, but there are also a few that hint at their anticancer activity, heralded an unprecedented and widespread interest in these plants. In this study, aiming to contribute by providing new knowledge on the evaluation of antioxidant and anticancer properties of this ethnobotanically valuable plant with its phytochemicals substantiated by LC-HRMS, six extracts prepared from *H. androsaemum* from Turkey were investigated. Accordingly, LM was found as the richest extract among them with 23 different constituents in it. On top of that, it had the highest total phenolic content. Expectedly, LM was found to be the strongest antioxidant mainly due to its rich phenolic content. Regarding the anticancer properties, FM was found to be significantly the most effective on apoptosis induction on PC-3 cell lines, also the strongest cytotoxic on PC-3 and Hep G2 cell lines among the six studied extracts. The anticancer effect of this extract may be due to quillaic acid, which is a triterpenoid, and was shown to have significant apoptosis induction ability and cytotoxic effect on different cell lines by different studies. In drawing things to close, being traditionally used against several problems, the evidence provided with this study point to the fact that *H. androsaemum* could be a very promising plant in the prevention of cancer.

## Contribution of authors

Nurdan Yazıcı Bektas: investigation, methodology, reviewing, software. Ezgi Ersoy: writing-original draft, conceptualization, methodology. Mehmet Boga: investigation, conceptualization, methodology, supervision, writing-reviewing and editing. Tugce Boran: investigation. Ercan Cinar: investigation. Gul Ozhan: supervision. Ahmet Ceyhan Goren: supervision, validation. Esra Eroglu Ozkan: conceptualization, methodology, visualization, writing-reviewing, supervision and editing.
